# P-82. Doxycycline versus other oral antibiotics for the treatment of gram-positive bone and joint infections

**DOI:** 10.1093/ofid/ofae631.289

**Published:** 2025-01-29

**Authors:** Joseph Corbino, Tyler Martin, Manish Patel, Rebecca Andruski, Sheena Kandiah, Marcos Schechter, Bruce Aldred

**Affiliations:** Rhode Island Hospital , Providence, Rhode Island; Archbold Medical Center, TALLAHASSEE, Florida; Grady Health System, Atlanta, Georgia; Grady Memorial Hospital, Atlanta, Georgia; Emory, Atlanta, Georgia; Emory University School of Medicine, Atlanta, GA; Emory University, Atlanta, Georgia

## Abstract

**Background:**

The OVIVA trial demonstrated oral agents with high bioavailability after one week of IV treatment is non-inferior in treatment of bone and joint infections (BJI) compared to continued IV therapy. Oral therapy significantly reduced rates of IV catheter complications and hospital length of stay. Certain highly bioavailable oral options with coverage for gram-positive organisms were well studied in this phase III trial, such as linezolid (LZD) and trimethoprim/sulfamethoxazole (TMP/SMX), with doxycycline representing a small proportion. LZD and TMP/SMX exceeded their respective Clinical and Laboratory Standards Institute (CLSI) breakpoints and MIC_90_ values in the bone. Doxycycline achieves concentrations of 3 mcg/mL in the bone but may not reach upper concentrations of susceptibility per the CLSI breakpoint (≤ 4 mcg/mL). LZD and TMP/SMX penetrate the bone well, but adverse effects may make prolonged therapy difficult to tolerate compared to doxycycline.

**Methods:**

A retrospective chart review was conducted of individuals ≥ 18 years prescribed doxycycline or either LZD or TMP/SMX for the treatment of a presumed or confirmed gram-positive BJI within the Grady Health System between January 1, 2021, and June 30, 2022. Individuals were included if they were referred to an ID provider within our Transitions of Care clinic and received oral antibiotics for ≥1 week on discharge. The composite primary outcome was suggested or performed amputation, OR debridement, all-cause mortality, or re-initiation of antibiotics. Secondary outcomes include rate of primary therapy completion and incidence of adverse effects.

**Results:**

77 patients met inclusion for the study, doxycycline (n=33) and LZD or TMP/SMX (n=44). There was no significant difference in the primary outcome at 9 months between either group (10 vs 12, p=0.77). Loss to follow-up drove incompletion rates of primary antibiotic therapy in both groups. Laboratory adverse effects were more common in the LZD or TMP/SMX group compared to doxycycline.

**Conclusion:**

Doxycycline may be associated with a favorable adverse effect profile compared to LZD or TMP/SMX. The efficacy of doxycycline compared to LZD or TMP/SMX should be considered exploratory with larger studies conducted to highlight its potential place in practice.

**Disclosures:**

**All Authors**: No reported disclosures

